# Research on the influencing factors of promoting flipped classroom teaching based on the integrated UTAUT model and learning engagement theory

**DOI:** 10.1038/s41598-024-66214-7

**Published:** 2024-07-02

**Authors:** Yufan Pan, Wang He

**Affiliations:** School of International Trade and Economics, Jiangxi University of Finance and Economics, Nanchang, 330013 China

**Keywords:** Flipped classroom, UTAUT, Learning engagement, Learning capability, 4C skills, Human behaviour, Information technology, Statistics

## Abstract

With the rapid advancement of educational technology, the flipped classroom approach has garnered considerable attention owing to its potential for enhancing students’ learning capabilities. This research delves into the flipped classroom teaching methodology, employing the Unified Theory of Acceptance and Use of Technology (UTAUT), learning engagement theory, and the 4C skills (comprising communication, collaboration, creativity, and critical thinking) to investigate its effects on learning capabilities. The research surveyed 413 students from three universities in Jiangxi Province, employing stratified random sampling. SPSS 24.0 and Amos were used for structural equation modeling and hypothesis testing analysis. The findings indicate that: (1) Performance expectancy, effort expectancy, and peer influence significantly enhance students’ learning engagement in the flipped classroom. (2) Students’ learning engagement in the flipped classroom notably promotes their learning capabilities. (3) Performance expectancy, effort expectancy, and peer influence can significantly boost learning capabilities by increasing learning engagement. (4) Personality traits significantly moderate the effect of peer influence on learning engagement, highlighting the crucial role of individual differences in learning. (5) The level of students’ learning engagement is differentially influenced by performance expectancy and peer influence across various academic disciplines. Ultimately, this research provides valuable insights for educational policymakers and guides improvements in teaching practices, collectively advancing educational quality and equity.

## Introduction

With the rapid development of technology and continuous innovation in educational philosophy, the flipped classroom, as a cutting-edge teaching method, has garnered widespread attention globally. This teaching approach was first proposed by two high school teachers, Jonathan Bergmann and Aaron Sams, in 2007, and has quickly gained widespread application at all levels of education^1^. The core concept of the flipped classroom lies in upending the traditional teaching model, extending students’ learning activities from the classroom to outside, thereby transforming the classroom into a hub for deep learning and practical activities. As such, exploring the impact of flipped classroom teaching on students’ learning abilities constitutes an important area of research. Currently, scholars both domestically and internationally focus their research on flipped classroom teaching primarily in the following two areas:

One aspect concerns studies on the implementation effects of the flipped classroom model. Numerous studies have confirmed its significant role in promoting students’ active participation, enhancing learning motivation, and improving academic performance. In biology instruction, Flores-González and Flores-González^2^ found that the flipped classroom fosters self-regulated learning and active engagement among students. Within an online educational environment, Cuetos^3^ research indicates that the flipped classroom model elevates university students’ learning motivation and academic achievement. In the field of English teaching, Dewi et al.^4^ study also revealed that students hold a positive attitude towards the new teaching mode of the flipped classroom. The flipped classroom provides students with more personalized and autonomous learning opportunities, allowing learning to align more closely with individual learning styles and paces^5,6^. Additionally, the impact of the flipped classroom on students’ academic success has garnered considerable attention. Semab and Naureen^7^ research demonstrates the positive effect of the flipped classroom model in enhancing students’ academic achievements. Studies by Roehl et al.^8^ and Tucker^9^ emphasize that the flipped classroom promotes the cultivation of students’ practical application abilities by reallocating classroom time for in-depth discussions, problem-solving, and hands-on activities.

Another area of research pertains to effective flipped classroom teaching design and the challenges it faces. Effective teaching design serves as the cornerstone of a successful flipped classroom. Nicholas^10^ underscores multiple teaching factors to consider when implementing a flipped classroom in graduate education. Meanwhile, Yang et al.^11^ designed a flipped classroom teaching model based on blended learning and experimentally verified the model’s effectiveness in enhancing students’ academic performance and level of learning engagement. For STEM education, Shofiyah et al.^12^ proposed a flipped classroom teaching template grounded in the 5E model, the validity and reliability of its content and structure have also been verified.

The research findings from the two aforementioned aspects provide rich references and insights for this research, yet there are two gaps in existing research. On the one hand, although the flipped classroom teaching method has been widely studied, few studies have explored the impact of the flipped classroom on enhancing 4C skills (communication, collaboration, creativity, and critical thinking), using the 4C skills as a measure of learning capability. On the other hand, despite the general agreement in previous studies on the positive effects of the flipped classroom on learning outcomes, few have investigated the differing impacts of personality and subject differences in flipped classroom teaching.

Therefore, this research seeks to answer the following questions:

Question 1: What are the key factors that affect students’ learning engagement in the flipped classroom teaching method?

Question 2: Does learning engagement further influence students’ 4C learning capabilities?

Question 3: Do individual differences (such as personality) and subject differences have a moderating effect between the relevant variables in this research?

To answer these questions, this research combines the UTAUT model, learning engagement theory, and the 4C skills analysis framework to construct a comprehensive research model. By collecting data from 413 students from three universities in Jiangxi Province, this research utilizes structural equation modeling for data analysis to unveil the complex relationships between performance expectancy, effort expectancy, peer influence, learning engagement, and learning capability in the flipped classroom.

The significance of this research lies not only in illustrating the specific impact of the flipped classroom on students’ learning capabilities through empirical analysis, providing a scientific basis for the further promotion and application of this teaching model, but also in deepening the understanding of the essence of the flipped classroom teaching model by integrating multiple theoretical frameworks. Furthermore, the research findings will provide strong support for educational policy formulation and teaching practice improvement, collectively advancing educational quality and equity.

Compared to existing research, the innovation of this research is primarily reflected in model construction and variable setting. Firstly, while preserving the core variables of the UTAUT model, this research innovatively integrates learning engagement theory and the 4C skills framework, offering a new perspective for understanding the interactions of various factors in the flipped classroom and their impact on learning capabilities. Secondly, in terms of variable setting, this research includes personality traits and subject differences as moderating variables in the analysis and utilizes 4C skills as a measure of learning capabilities, which is a novel approach in the field of flipped classroom teaching research.

In the following chapters, this research will conduct a literature review, derive research hypotheses, describe the survey process, perform model inspection, and finally discuss and present research implications.

## Literature review

### Theory and application of the UTAUT

The UTAUT model, proposed by Venkatesh et al.^13^, integrates key variables from multiple theoretical models, including the Theory of Reasoned Action (TRA), Theory of Planned Behavior (TPB), Technology Acceptance Model (TAM), Motivational Model (MM), Combined TAM and TPB (C-TAM), and Innovation Diffusion Theory (IDT). Its aim is to provide a more comprehensive and accurate framework to explain and predict users’ acceptance behavior towards new technologies.

The dimensions of the UTAUT model have demonstrated strong explanatory power in technology adoption research across multiple domains, particularly in education. The model has been applied to studies on the acceptance behavior of technologies such as Massive Open Online Courses (MOOCs) and AI chatbots. Li and Zhao^14^ combined the UTAUT model with Social Presence Theory to analyze factors influencing students’ continued use of MOOCs, finding that the UTAUT model positively affects students’ satisfaction and intention to continue using MOOCs. Meanwhile, Tian et al.^15^ utilized both UTAUT and ECM models to explore Chinese graduate students’ acceptance and utilization of AI chatbot technology, discovering that “confirmation” and “satisfaction” from the ECM model have a greater impact on user behavior than the UTAUT model.

The flipped classroom, as an innovative teaching model in higher education, has garnered significant attention regarding its application effects and influencing factors. In this domain, both Alyoussef^16^, Agyei and Razi^17^ have conducted in-depth explorations utilizing the UTAUT model. Alyoussef^16^ revealed the central mediating role of perceived usefulness and perceived ease of use in students’ acceptance of the flipped classroom, using a sample of students from a university in Saudi Arabia. This finding not only indicates students’ positive attitude towards the flipped classroom but also further confirms the positive role of this teaching model in enhancing learning outcomes. Agyei and Razi^17^ enriched the UTAUT model by introducing variables such as experience expectancy, parent-school involvement, perceived behavioral control, and self-efficacy to deeply analyze high school students’ acceptance of using online resources for flipped classroom learning. Their results showed that performance expectancy, effort expectancy, parent-school involvement, students’ self-efficacy, and experience expectancy all significantly positively impact students’ willingness to learn. However, perceived behavioral control did not show a significant effect in their research.

In this research, we focus on the college student population whose social connections are primarily reflected in peer relationships. Therefore, we choose to represent the social influence element in the UTAUT model with the peer influence, aiming to be more aligned with the actual situation of this specific group. Meanwhile, considering the widespread popularity of technology in modern higher education, we do not consider facilitating conditions as a core factor in our research, although this does not imply that this element can be ignored in all environments.

### Learning engagement theory

Learning engagement theory, initially proposed by Fredricks et al.^18^ in the field of educational psychology, aims to delve into students’ cognitive, emotional, and behavioral engagement exhibited during the learning process. This theory focuses on assessing the enthusiasm and depth of students’ participation in learning activities, encompassing three key dimensions: cognitive engagement, emotional engagement, and behavioral engagement.

In the process of deeply exploring the theory of learning engagement, researchers have conducted extensive studies targeting different educational environments and learning formats. The issue of learning engagement is particularly prominent in the field of MOOCs and online learning. Although MOOCs have attracted a large number of learners due to their openness and flexibility, the low completion rate has always been one of the challenges they face^19^. Studies have shown that students’ intrinsic motivations (such as interest) and extrinsic motivations (such as perceived knowledge value) have a significant impact on their learning engagement in MOOCs. From the perspective of self-determination theory, Lan and Hew^20^ research adopted a mixed-method approach to investigate learning engagement in MOOCs. The study found that perceived ability and emotional engagement have a significant impact on students who complete MOOCs, and different dimensions of learning engagement can predict learners’ perceived learning effectiveness. Meanwhile, in the online learning environment, significant changes have occurred in the way students interact with teachers and peers, which requires educators to pay more attention to cultivating students’ autonomous learning abilities, computer and network skills, and online communication abilities to promote their learning engagement^21^.

### Related research on the 4C skills analysis framework

Voogt et al.^22^ emphasized the importance of 4C skills in 21st-century education in their study. These skills include Communication, Collaboration, Creativity, and Critical Thinking, which focus on cultivating students’ comprehensive literacy to adapt to the complex needs of modern society. Especially when measuring learning capability, the 4C skills provide a comprehensive and appropriate framework.

In the context of exploring the improvement of learning abilities, integrating these four skills—critical thinking, communication, collaboration, and creativity—into the learning process is particularly critical. Specifically, critical thinking skills enable learners to identify true and false information and adapt to environmental changes^23^. Communication skills, including effective speaking, listening, and writing, are key to improving interpersonal efficiency^24^. Collaboration emphasizes working together in a team environment, facilitating knowledge sharing and problem-solving^25^. Innovation ability is the key to gaining an advantage in modern social competition, requiring continuous learning, challenging traditional concepts, and maintaining sensitivity to new technologies^26^. By integrating these abilities, the learning process can be more comprehensive, improving flexibility and effectiveness in responding to various challenges.

Although previous studies have explored the role of the flipped classroom model in promoting student collaboration, criticism, and innovation abilities^9,27^, there is still a lack of in-depth exploration of the specific impact and mechanism of communication ability in this model. Therefore, this research aims to comprehensively integrate 4C abilities and deeply explore the overall impact of the flipped classroom teaching model on these abilities.

This research combines the UTAUT model, learning engagement theory, and the 4C theory, selecting Performance expectancy (PE), Effort expectancy (EEX), Peer influence (PI), Learning engagement (ENGA), and Learning capability (SKIL) as research constructs to explore the impact mechanism of flipped classroom teaching on college students’ learning capability. The specific definitions of the variables are shown in Table 1:

**Table 1:** Operational definitions of research variables.

Variable	Definition	Literature source
Performance expectancy (PE)	University students’ expectations for enhancing learning outcomes through participation in the flipped classroom teaching model	Venkatesh et al.^13^, Chen and Wu^36^, Clark^30^, Riddle and Gier^29^, Singh^28^, Plageras et al.^58^
Effort expectancy (EEX)	University students’ perceived difficulty in mastering the skills and knowledge required for the flipped classroom	Chen and Wu^36^, Jia et al.^31^, Plageras et al.^58^
Peer influence (PI)	In the flipped classroom environment, students’ perspectives on using this teaching model and its influence on individual choices	Chen and Wu^36^, Kissi et al.^59^, Ruiz^32^, Plageras et al.^58^
Learning engagement (ENGA)	The level of active participation of university students in the flipped classroom, encompassing three aspects: cognitive engagement (CE), emotional engagement (EEN), and behavioral engagement (BE)	Zimmerman^33^, Martin and Bolliger^35^, Tang and Hew^60^
Learning capability (SKIL)	The ability of students to acquire and apply knowledge and skills through the flipped classroom, including four aspects: critical thinking (CT), communication (CS), creativity (CREA), and collaboration (CSK)	Voogt et al.^22^, Tucker^9^, Zainuddin and Halili^27^, Sari and Wardhani^24^, Nuraini et al.^26^,Solichah et al.^23^, Pratama et al.^25^

## Hypothesis derivation

### The relationship between performance expectancy, effort expectancy, peer influence, and learning engagement

Singh^28^, Riddle and Gier^29^ and Clark^30^ and other scholars have found that flipped classrooms can improve students’ test scores and course engagement by replacing traditional lectures with micro-lectures and activity-based learning strategies, thus demonstrating that performance expectancy have a significant positive impact on students’ engagement in learning. The fully online flipped classroom model was more effective than the online flipped model in supporting student behavioral engagement, suggesting the importance of effort expectancy in an online environment^31^. Ruiz^32^ study demonstrated that integrating interactive technology and peer instruction into a flipped classroom can positively affect student engagement, i.e., the importance of peer influence in enhancing student engagement and learning. Based on the above, the following hypotheses are proposed in this research:

#### H1

Performance expectancy has a significant positive impact on students’ learning engagement.

#### H2

Effort expectancy has a significant positive impact on students’ learning engagement.

#### H3

Peer influence has a significant positive impact on students’ learning engagement.

### The relationship between learning engagement and learning capability

Learning Engagement Theory emphasizes the cognitive, emotional, and behavioral engagement of students in the learning process and these factors are believed to positively influence the enhancement of learning capabilities. Zimmerman^33^ states that when students believe they can succeed in a learning task, they are more likely to invest more energy and effort, which promotes learning capabilities. Pekrun et al.^34^ explored the relationship between students’ engagement in learning and learning competence from an emotional perspective, which further supports the positive link between engagement in learning and increased learning competence. Martin and Bolliger^35^ noted that student engagement in learning directly impacts online learning outcomes, improves student performance in online programs and is considered an important factor in measuring teaching quality. Taken together, these studies suggest that learning engagement can have a significant positive impact on students’ learning capabilities by influencing their self-efficacy, motivation, learning strategies, and emotional engagement. Based on the above, this research proposes the following hypotheses:

#### H4

Students’ learning engagement has a significant positive impact on learning capability.

### The mediating role of learning engagement

According to the aforementioned literature, it is evident that performance expectancy, effort expectancy, and peer influence have a positive effect on the enhancement of learning capabilities through learning engagement. This is supported by studies from Chen and Wu^36^, Buabeng-Andoh^37^, Kuo et al.^38^, Zimmerman^33^, Pekrun et al.^34^, and Martin and Bolliger^35^. Additionally, research by Jamaludin and Osman^39^, Wang^40^, and Nerantzi^41^also corroborate the notion that performance expectancy, effort expectancy, and peer influence impact learning capabilities through learning engagement. Based on this information, the current study proposes the following hypotheses:

#### H5

Performance expectancy has a significant positive impact on learning capability through students’ learning engagement.

#### H6

Effort expectancy has a significant positive impact on learning capability through students’ learning engagement.

#### H7

Peer influence has a significant positive impact on learning capability through students’ learning engagement.

### Moderating variables

#### Personality

Personality is a relatively stable individual difference in behavior, emotion, and cognition exhibited by an individual that encompasses traits, habits, attitudes, and values. Eysenck and Eysenck and Eysenck^42^ Proposed the introversion–extraversion theory to explain and describe individual personality differences, which divides human personality into two types: introverted and extroverted. Chuang et al.^43^, Kim et al.^44^ and other scholars explored the differences in classroom performance of students with different personality traits in a flipped classroom, with Wang et al.^45^ noted that students with moderate openness performed best in flipped classrooms, while students with high openness performed best in online learning situations. However, there are relatively few studies on personality as a moderating variable in the UTAUT model because UTAUT focuses primarily on technology acceptance and use behaviors, while individual differences, including personality, are usually more prominent in other models. Combined with the traits of the subjects under study, introverted students may be more inclined to show higher learning engagement in independent learning environments, while extroverted students may be more adept at collaborating with peers, therefore, the variable of personality is added as a moderating variable in this study. Based on the above, the following hypotheses are proposed in this research:

##### H8A

Personality has a moderating effect between performance expectancy and learning engagement.

##### H8B

Personality has a moderating effect between effort expectancy and learning engagement.

##### H8C

Personality has a moderating effect between peer influence and learning engagement.

##### H8D

Personality has a moderating effect between learning engagement and learning capability.

#### Subject

Based on the perspective of individual differences, subject differences may have different impacts on students’ engagement and learning capabilities in the flipped classroom environment. Liu et al.^46^ explored the impact of a flipped classroom integrating subjects on student learning capabilities in different health professional fields. Meanwhile, Fan^47^ explored the application of social influences, school motivation, and gender differences in educational psychology and found that disciplinary background may influence student motivation and engagement. Students may exhibit different learning preferences and strategies in humanities and social sciences and science and technology disciplinary contexts. Meanwhile, subject differences in the UTAUT model have been investigated by focusing on the similarities and differences in individuals’ acceptance of technology in different subject areas. Therefore, this research proposes the following hypotheses about the moderating effects of disciplinary differences:

##### H9A

Discipline has a moderating effect between performance expectancy and learning engagement.

##### H9B

Subject has a moderating effect between effort expectancy and learning engagement.

##### H9C

Subject has a moderating effect between peer influence and learning engagement.

##### H9D

Subject has a moderating effect between learning engagement and learning capabilities.

**Figure 1:**
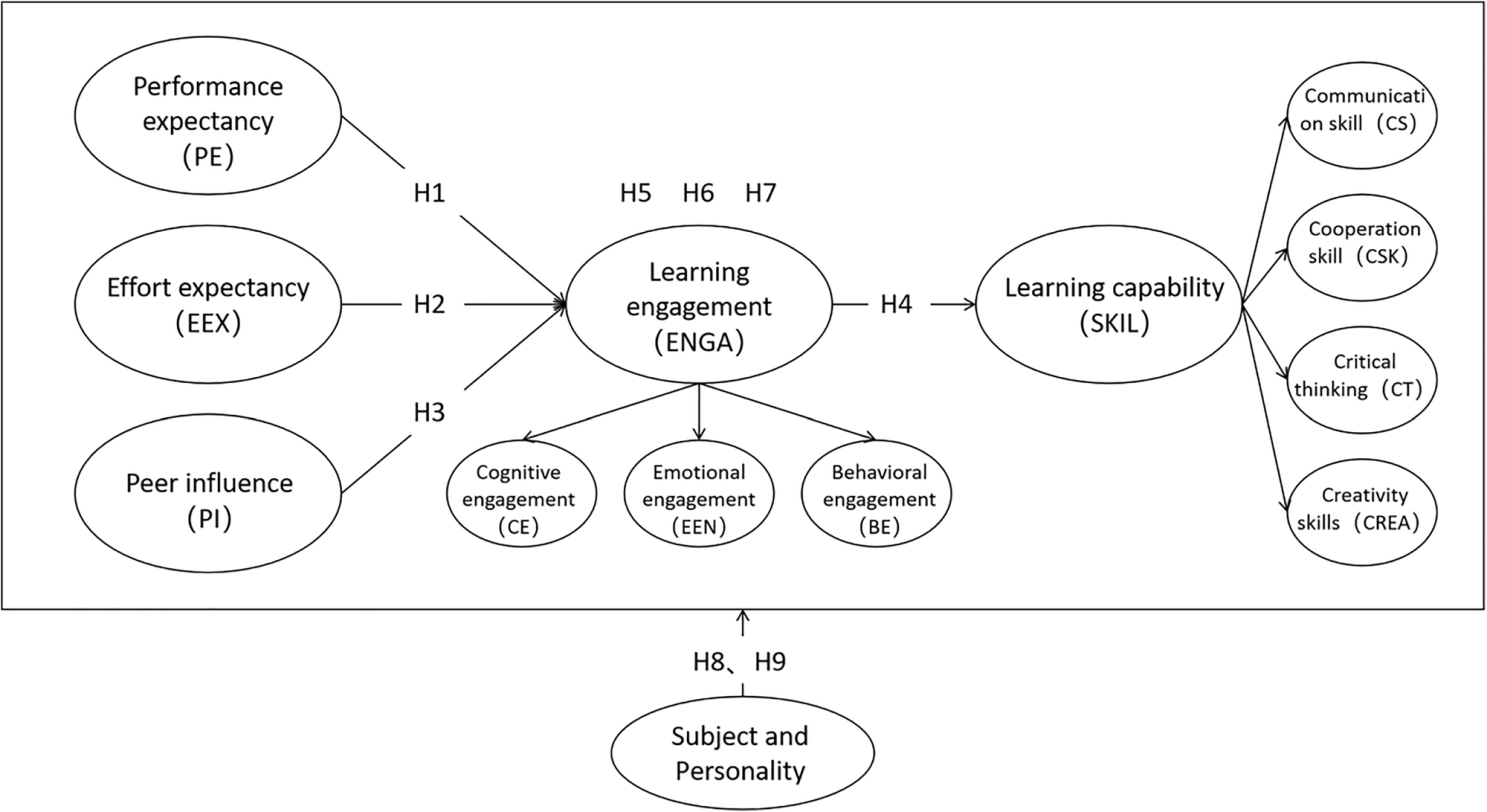
Theoretical model of flipped classroom teaching to enhance students’ learning capability.

Taking the above into account, a theoretical model can be constructed about the enhancement of students’ learning capabilities by flipped classroom teaching, which is shown in Fig. 1.

## Data collection

### Collection method

This research employed a questionnaire survey for data collection. The survey was conducted from November to December 2023, targeting students who had participated in flipped classroom learning in three universities in Jiangxi Province. To ensure the representativeness and breadth of the sample, the research team adopted a stratified random sampling method. A total of 450 eligible students were selected as survey respondents. This sampling strategy aimed to ensure that the sample comprehensively reflected the characteristics and opinions of students at different levels, thereby enhancing the reliability and validity of the research.

During the survey process, a detailed questionnaire was distributed to each participating student, and they were offered a cash incentive to encourage honest and thoughtful responses. Through this approach, we hoped to gather high-quality data that truly reflected students’ flipped classroom experiences, providing a solid foundation for subsequent analysis and research.

A total of 450 questionnaires were distributed, with 418 returned (93% response rate) and 413 valid questionnaires ultimately obtained (99% validity rate). Throughout the questionnaire distribution process, we strictly adhered to ethical principles in academic research, particularly regarding obtaining informed consent from participants. All students participating in this research were clearly informed about the purpose, methodology, potential risks, and their rights related to this research.

### Collection instruments

The questionnaire employed a 7-point Likert scale, with the numbers 1, 2, 3, 4, 5, 6, and 7 representing “strongly disagree,” “disagree,” “somewhat disagree,” “neutral,” “somewhat agree,” “agree,” and “strongly agree,” respectively. “Strongly disagree” indicates that the situation described in the item completely contradicts reality, “strongly agree” indicates complete agreement with reality, and “neutral” indicates a middle ground. The research tool used in this research consists of six parts. The first part is a questionnaire on student background variables (including subject, personal personality, etc.). The second part is a performance expectancy questionnaire, adopting questionnaire content designed by Devisakti and Ramayah^48^ and others for performance expectancy. It focuses on specific measurement items for university students’ performance expectancy of flipped classrooms and consists of 4 questions. The third part is an effort expectancy questionnaire, utilizing content designed by Zou et al.^49^ and others for effort expectancy. It aims to measure specific aspects of university students’ effort expectancy in flipped classrooms and includes 3 questions. The fourth part is a peer influence questionnaire, based on content designed by Zhonggen and Xiaozhi^50^, Khlaisang et al.^51^, and others. It measures specific aspects of peer influence in flipped classrooms for university students and comprises 3 questions. The fifth part is a learning engagement questionnaire, using content designed by Qureshi et al.^52^. This section includes behavioral, emotional, and cognitive engagement as sub-dimensions and aims to measure specific aspects of university students’ learning engagement in flipped classrooms with 12 questions. The sixth part is a personal capability enhancement questionnaire, employing content designed by Arshad and Akram^53^, C.-H. S. Liu^54^, Baruch and Lin^55^ and others. It includes sub-dimensions such as communication skills, cooperation skills, innovation skills, and critical thinking skills, aiming to measure specific aspects of personal capability enhancement in university students after learning in flipped classrooms, with 16 questions.

### Demographic analysis

In this research, data collection resulted in the recovery of 418 questionnaires. After discarding 5 invalid samples, 413 valid samples remained. The basic characteristics are shown in Table 2. The primary data collected in this research includes subjects and personal personality traits. Among the 418 surveyed students, 199 were from science and engineering, accounting for 48.18% of the total; 214 were from humanities and social sciences, accounting for 51.82%. In terms of personality traits, 201 students were introverted, accounting for 48.67% of the total, while 212 students were extroverted, representing 51.33% of the total.

**Table 2:** Frequency table of demographic analysis (N = 413).

Variable	Variable label	Frequency	Percentage
Subject	Science and engineering	199	48.18 percent
	Humanities and social science	214	51.82 percent
Personality	Introverted (personality)	201	48.67 percent
	Export-oriented (personality)	212	51.33 percent

## Model inspection and results

In this research, SPSS 24.0 and AMOS software were used to perform structural equation modeling analysis on the data to explore the impact of flipped classroom teaching on students’ learning abilities. The analysis primarily consists of two parts: the measurement model (including reliability testing, convergent validity testing, and discriminant validity testing) and the structural model (including model fit analysis, path analysis, mediation effect analysis, and moderation effect analysis).

### Measurement model analysis

#### Questionnaire reliability test

Table 3 presents the internal consistency of the questionnaire dimensions. The internal consistency (Cronbach’s α) of all dimensions is higher than 0.7, indicating good reliability of the questionnaire sample data. All items will be retained for subsequent analysis.

**Table 3:** Questionnaire reliability.

Dimensions	Cronbach’s α
PE	0.89
EEX	0.84
PI	0.86
CE	0.91
EEN	0.84
BE	0.87
CS	0.88
CT	0.9
CREA	0.89
CSK	0.85

#### Convergence validity test

Table 4 presents the standardized factor loadings of each measurement item, as well as the composite reliability and average variance extracted (AVE) for each dimension. The standardized factor loadings range from 0.648 to 0.851, the composite reliability falls between 0.806 and 0.913, and the AVE is between 0.58 and 0.683. These values meet the criteria established by Fornell and Larcker^56^, indicating good convergence validity of the research.

**Table 4:** Convergence validity.

Dimensions	Indicators	Std	CR	AVE
PI	PI01	0.833	0.855	0.664
	PI02	0.804		
	PI03	0.807		
EEX	EEX01	0.797	0.842	0.64
	EEX02	0.824		
	EEX03	0.778		
PE	PE01	0.817	0.891	0.671
	PE02	0.8		
	PE03	0.813		
	PE04	0.845		
CE	CE01	0.812	0.913	0.677
	CE02	0.807		
	CE03	0.851		
	CE04	0.813		
	CE05	0.831		
EEN	EEN01	0.819	0.84	0.636
	EEN02	0.761		
	EEN03	0.812		
BE	BE01	0.804	0.875	0.635
	BE02	0.776		
	BE03	0.799		
	BE04	0.809		
CS	CS01	0.802	0.883	0.653
	CS02	0.792		
	CS03	0.819		
	CS04	0.819		
CT	CT01	0.813	0.896	0.683
	CT02	0.825		
	CT03	0.826		
	CT04	0.842		
CREA	CREA01	0.809	0.889	0.666
	CREA02	0.813		
	CREA03	0.826		
	CREA04	0.817		
CSK	CSK01	0.820	0.877	0.641
	CSK02	0.792		
	CSK03	0.787		
	CSK04	0.804		
ENGA	CE	0.747	0.806	0.58
	EEN	0.689		
	BE	0.648		
SKIL	CS	0.79	0.876	0.639
	CT	0.798		
	CREA	0.783		
	CSK	0.826		

### Discriminant validity

This research employed the Average Variance Extracted approach to evaluate the discriminant validity. In order to have sufficient discriminant validity, each construct’s square root of the AVE should be greater than the correlation coefficients between the constructs, according to Fornell and Larcker^56^. Table 5 displays the data demonstrating that the square roots of the AVEs for each of the components are higher than the associated correlation coefficients. The good discriminant validity of the model is confirmed by this finding. Make sure every research concept is unique and not just a reflection of other variables in the model by using discriminant validity.

**Table 5:** Discriminant validity.

	AVE	PI	EEX	PE	ENGA	SKIL
PI	0.664	**0.815**				
EEX	0.640	0.577	**0.800**			
PE	0.671	0.635	0.565	**0.819**		
ENGA	0.580	0.630	0.645	0.655	**0.762**	
SKIL	0.639	0.725	0.673	0.673	0.706	**0.799**

A footnote to explain the [bold] values has been added to table [5]. Please could you confirm the footnote? Or, confirm that we should remove the [bold] and the footnote from the table?

### Structural model analysis

#### Model fit

The values of the model fit indices for the structural equation model fall within an acceptable range, as Table 6 shows. Values fewer than 3 or 5 (depending on the criterion employed) are generally considered suggestive of a good fit, therefore the χ^2^/DF value of 1.002 is desirable. A excellent match is shown by the RMSEA value of 0.002, which is significantly less than the typical threshold of 0.08. A good model fit is indicated by the SRMR value of 0.038, which is less than the suggested maximum of 0.08. Both the CFI and the TLI values of 0.999 and 0.999 are near to 1, indicating a very good fit to the data. A good model fit is further confirmed by the values of the GFI and AGFI, which are 0.938 and 0.933, respectively, above the generally recognized criterion of 0.90. Together, these fit indices imply that the structural equation model provides a good match to the data, indicating that the model accurately captures the observed data.

**Table 6:** Model fit.

Model fit	Criteria	Model fit of the research model
model fit	Tolerable range	
MLχ^2^ chi-square value	The smaller the better	652.585
degree of freedom	The larger the better	651.000
Normed Chi-sqr (χ^2^ /DF) Chi-square value/degrees of freedom	1 < χ^2^/DF < 3	1.002
Root Mean Square of Approximation Error	< 0.08	0.002
Standardized residual root mean square	< 0.08	0.038
Tucker-Lewis Indicator (NNFI)	> 0.9	0.999
Comparison of Fit Indicators	> 0.9	0.999
Goodness-of-fit indicator	> 0.9	0.938
Adjusted Goodness-of-Fit Indicator	> 0.9	0.933

#### Path analysis

**Figure 2:**
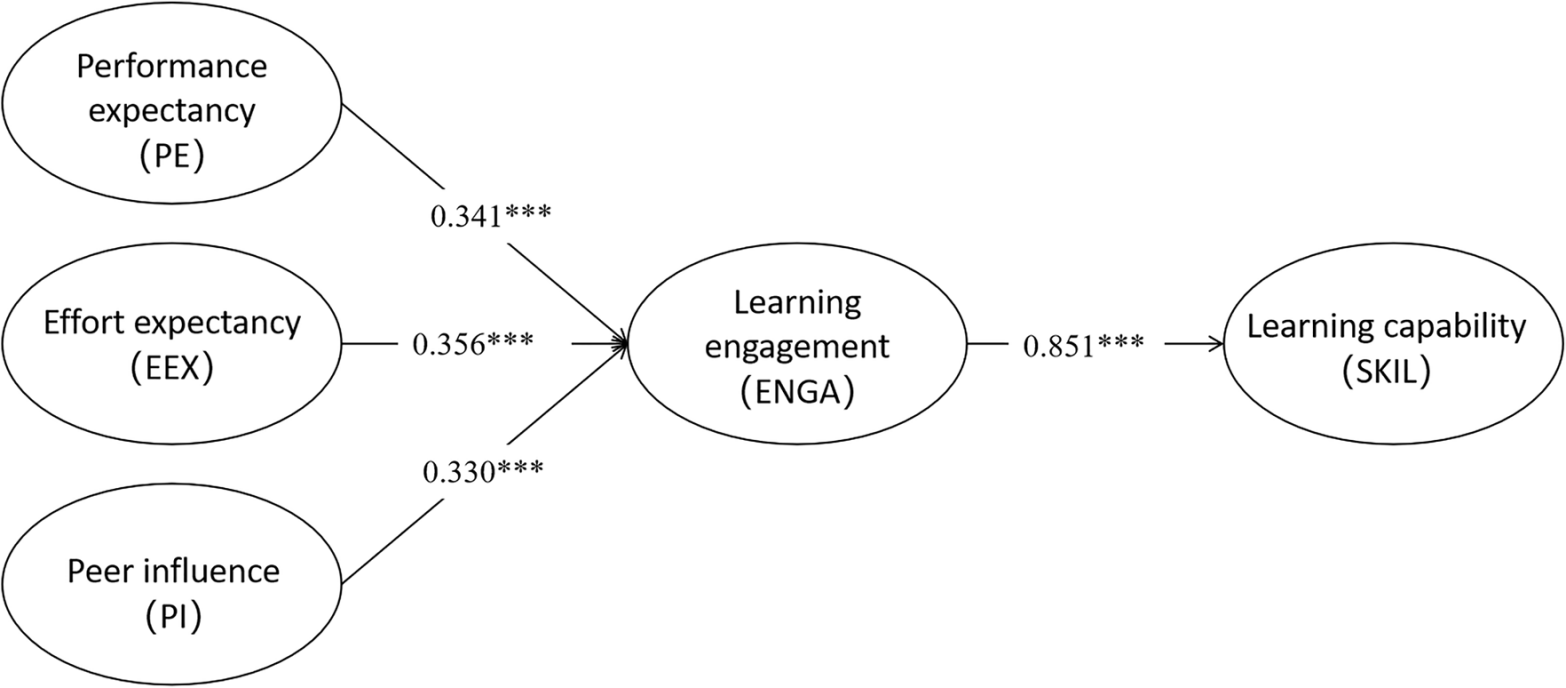
Path analysis results. Note: *p < 0.05;**p < 0.01;***p < 0.001, All present standardized regression coefficients.

According to the path coefficient analysis results presented in Table 7 and Fig. 2, Performance Expectancy (PE) (b = 0.244, *p* < 0.001), Effort Expectancy (EEX) (b = 0.284, *p* < 0.001), and Peer Influence (PI) (b = 0.242, *p* < 0.001) all have a significant positive impact on Learning Engagement (ENGA). Furthermore, Learning Engagement (ENGA) also significantly and positively affects the Learning Capability (SKIL) (b = 0.838, *p* < 0.001). Therefore, hypotheses H1, H2, H3, and H4 are supported.

**Table7:** Regression coefficients.

DV	IV	Unstd	S.E	Unstd./S.E	*p*-value	Std	R^2^
ENGA	PE	0.244	0.046	5.341	***	0.341	0.766
	EEX	0.284	0.049	5.814	***	0.356	
	PI	0.242	0.045	5.359	***	0.330	
SKIL	ENGA	0.838	0.082	10.219	***	0.851	0.725

DV stands for the dependent variable, IV stands for the independent variable, Unstd stands for unstandardized, S.E. stands for standard error, *p*-value stands for *p*-value, Std. stands for standardized regression coefficients, and R^2^ stands for the amount of explainable variance.

Furthermore, the findings show that whereas learning engagement accounts for 72.5% of the variance in the improvement of learning skills, performance expectancy, effort expectancy, and peer influence collectively account for 76.6% of the variance in learning engagement. These results underline the significance of Performance Expectancy, Effort Expectancy, and Peer Influence in determining Learning Engagement and validate the research hypothesis. Additionally, they attest to the importance of learning engagement in improving learning capability. This emphasizes how important these factors are to the flipped classroom learning environment and how they all work together to enhance student learning capabilities.

### Mediation effect

Based on the indirect effects analysis in the mediation model shown in Table 8, it is noticed that the *p*-values are significant and the confidence intervals do not include 0 in all three mediated hypothesis paths (PI → ENGA → SKIL, EEX → ENGA → SKIL, and PE → ENGA → SKIL). This indicates that the mediation effects are valid in all cases. Specifically:

**Table 8:** Analysis of the indirect effects of the intermediary model.

Effect	Point estimate	product of coefficients			Bootstrap 1000 times	
					Bias-corrected 95 per cent	
		S.E	Z-Value	*p*-value	Lower bound	Upper bound
PI → ENGA → SKIL	0.204	0.047	4.384	***	0.116	0.301
EEX → ENGA → SKIL	0.238	0.047	5.024	***	0.152	0.337
PE → ENGA → SKIL	0.203	0.041	4.905	***	0.130	0.302

PE has a significant indirect effect on the enhancement of SKIL through ENGA, supporting Hypothesis H7. EEX has a significant indirect effect on the enhancement of SKIL through ENGA, supporting Hypothesis H6. PE has a significant indirect effect on the enhancement of SKIL through ENGA, supporting Hypothesis H5.

These findings demonstrate the critical role of ENGA as a mediator in the relationship between PE, EEX, PI, and the enhancement of SKIL. This supports the theoretical framework proposed in the research, highlighting the importance of these constructs in the context of flipped classroom learning environments.

### Moderating effect

When considering individual personality as a moderating variable, among the 413 respondents, there were 201 introverted students and 212 extroverted students. Table 9 presents the regression coefficient values for the two groups, showing the comparison of slope differences between them. Table 10 displays the moderation effect test of the model. Among the four cross-group comparisons of slopes, the path of PI → ENGA reaches a significant level, indicating that the moderation effect is partially established, thus confirming Hypothesis H8C. From the values in Table 9, it can be observed that the regression coefficient of PI → ENGA for introverted students is significantly higher than that of extroverted students.

**Table 9:** Regression estimates of moderating effects.

IV	DV	Personality								Subject							
		Introverted				export-oriented				science and engineering				humanities and social science			
		Estimate	S.E	z	*p*	Estimate	S.E	z	*p*	Estimate	S.E	z	*p*	Estimate	S.E	z	*p*
PE	ENGA	0.179	0.066	2.7	0.007	0.232	0.06	3.85	***	**0.616**	0.218	2.828	0.005	0.147	0.053	2.788	0.005
EEX	ENGA	0.266	0.074	3.617	***	0.257	0.059	4.351	***	0.267	0.079	3.388	***	0.218	0.071	3.07	0.002
PI	ENGA	**0.328**	0.071	4.601	***	0.132	0.05	2.654	0.008	0.151	0.058	2.629	0.009	**0.387**	0.083	4.645	***
ENGA	SKIL	0.909	0.108	8.449	***	0.857	0.162	5.299	***	0.8	0.137	5.823	***	0.811	0.104	7.763	***

A footnote to explain the [bold] values has been added to table [9]. Please could you confirm the footnote? Or, confirm that we should remove the [bold] and the footnote from the table?

**Table 10:** Difference in path coefficients of the regulation effects.

Model	Personality							Subject						
	Model fit				Nested model differences			Model fit				Nested model differences		
	NPAR	χ^2^	DF	χ^2^ /DF	ΔDF	Δχ^2^	*p*	NPAR	χ^2^	DF	χ^2^ /DF	ΔDF	Δχ^2^	*p*
Default	180	1317.000	1302	1.012				180	1351.538	1302	1.038			
PE→ ENGA	179	1317.353	1303	1.011	1	0.354	0.552	179	1358.088	1303	1.042	1	6.551	**0.010**
EEX→ ENGA	179	1317.008	1303	1.011	1	0.009	0.926	179	1351.743	1303	1.037	1	0.205	0.650
PI→ ENGA	179	1322.099	1303	1.015	1	5.099	**0.024**	179	1357.045	1303	1.041	1	5.507	**0.019**
ENGA→ SKIL	179	1317.066	1303	1.011	1	0.066	0.798	179	1351.541	1303	1.037	1	0.003	0.954

NPAR, number of parameters.

A footnote to explain the [bold] values has been added to table [10]. Please could you confirm the footnote? Or, confirm that we should remove the [bold] and the footnote from the table?

When considering the subject as a moderating variable, among the 413 respondents, there were 199 science and engineering students and 214 humanities and social science students. Table 9 shows the regression coefficient values for the two groups, representing the comparison of slope differences between them. Table 10 presents the moderation effect test of the model. Among the eight cross-group comparisons of slopes, the paths of PE → ENGA and PI → ENGA reach significant levels, indicating that the moderation effect is partially established, thus confirming Hypotheses H9A and H9C. From the values in Table 9, it can be seen that the regression coefficient of PE → ENGA for science and engineering students is significantly higher than that of humanities and social science students, while the regression coefficient of PI → ENGA for humanities and social science students is significantly higher than that of science and engineering students.

## Conclusion and discussion

### Main Research Findings and Conclusions

Based on Table 11, the main findings of this research are as follows:

1. The four main effect hypotheses, H1, H2, H3, and H4, are validated. Analyses confirming H1, H2, and H3 demonstrate that Performance Expectancy, Effort Expectancy, and Peer Influence significantly positively influence Learning Engagement, aligning with research by Singh^28^, Riddle and Gier^29^, Clark^30^, Jia et al.^31^, and Ruiz^32^. The validation of H4 shows that Learning Engagement significantly positively impacts Learning Capability, consistent with Pekrun et al.^34^, Zimmerman^33^, and Martin and Bolliger^35^.
2. The three mediation effect hypotheses, H5, H6, and H7, are supported. Analyses for H5, H6, and H7 indicate that Performance Expectancy, Effort Expectancy, and Peer Influence significantly and positively influence Learning Capabilities through Learning Engagement, aligning with findings by Jamaludin and Osman^39^, Wang^40^, Kobayashi^57^, and Nerantzi^41^.
3. The two moderating effect hypotheses, H8C, H9A, and H9C, are validated. Analysis for H8C shows a significant moderating effect of personality between Peer Influence and Learning Engagement, consistent with Wang et al.^45^. Analyses for H9A and H9C reveal significant moderating effects of the subject field on the paths from Performance Expectancy to Learning Engagement and Peer Influence to Learning Engagement, echoing Fan^47^.

**Table 11:** Presentation of research results.

Effect	Hypothesis	Hypothetical content	Decision
Main effect	H1	Performance expectancy has a significant positive impact on students’ learning engagement	Supported
	H2	Effort expectancy has a significant positive impact on students’ learning engagement	Supported
	H3	Peer influence has a significant positive impact on students’ learning engagement	Supported
	H4	Students’ learning engagement has a significant positive impact on their learning capability	Supported
Mediating effect	H5	Performance expectancy has a significant positive impact on learning capability through students’ learning engagement	Supported
	H6	Effort expectancy has a significant positive impact on learning capability through students’ learning engagement	Supported
	H7	Peer influence has a significant positive impact on learning capability through students’ learning engagement	Supported
Moderating effect	H8C	Personality has a moderating effect between peer influence and learning engagement	Supported
	H9A	Subject has a moderating effect between performance expectancy and learning engagement	Supported
	H9C	Subject has a moderating effect between peer influence and learning engagement	Supported

#### Main research conclusions

1. Performance expectancy, effort expectancy, and peer influence notably enhance students’ learning engagement in the flipped classroom teaching. Performance expectancy encourages students to set specific learning goals, work towards achieving them, and believe in their potential to excel academically, thereby increasing their focus and dedication to studies. At the same time, a clear understanding of the required effort makes students appreciate every moment in the flipped classroom, fully investing themselves in the learning experience. Moreover, peer influence is essential as students collaborate and interact, fostering a supportive learning environment that further fuels their motivation and boosts learning engagement.
2. Students’ learning engagement in the flipped classroom teaching significantly enhances their learning capabilities. Highly engaged students exhibit more active participation in class discussions, thereby refining their oral expression and improving their communication skills for smoother and more efficient interactions. Additionally, the flipped classroom frequently necessitates group work, fostering a sense of teamwork and collaboration among students. Through rigorous reflection and problem-solving exercises, students cultivate critical thinking abilities, allowing them to objectively and comprehensively analyze issues. Moreover, the flipped classroom underscores independent learning and exploration, thus motivating students to tackle problems from diverse perspectives and stimulating their innovative thinking and creativity. As a result, students who demonstrate high engagement in the flipped classroom achieve not only academic excellence but also substantial improvements in their communication, collaboration, critical thinking, and creativity skills.
3. Performance expectancy, effort expectancy, and peer influence can significantly enhance learning capability by increasing learning engagement. Clear performance expectancy fuels students’ motivation, keeping them laser-focused on learning tasks. To achieve better outcomes, students become proactive in communicating with peers and teachers, thus honing their communication skills. Additionally, they collaborate more with classmates to solve problems, strengthening their teamwork abilities. Effort expectancy helps students understand that to reach their learning goals, consistent effort is key. This realization sharpens their critical thinking and drives them to continuously refine their learning methods. In this journey, students also experiment with novel learning strategies, fostering creativity. Moreover, peer influence plays a pivotal role in shaping the learning environment. Mutual encouragement and imitation among peers create a positive atmosphere, prompting deeper engagement and significantly boosting students’ communication, collaboration, critical thinking, and creativity skills.
4. Personality plays a significant moderating role in the impact of peer influence on learning engagement, emphasizing the role of individual differences in learning. Our personality traits guide us in choosing our peers, often leading similar-minded students to form study groups. This, in turn, shapes the way peers influence each other and how engaged they are in learning through unique interaction styles. Additionally, our personalities determine our preferred learning methods. Extroverted students, for example, might enjoy learning through group discussions and hands-on activities, while introverted students might prefer solo research and quiet reading. These personality-driven choices further shape our attitudes, motivation, and how we handle learning challenges.
5. In various subject areas, students’ learning engagement is influenced to differing extents by performance expectancy and peer influence. The level of difficulty of the subjects and the students’ personal interests directly shape their performance expectancy. For instance, the abstract logic in science and engineering subjects may pose a challenge to students, influencing their expectations, while the memorization and comprehension in humanities and social sciences may be relatively easier, leading to more optimistic Performance Expectancy. Additionally, subject characteristics shape Peer Influence, as problem-solving in science and engineering subjects and text interpretation in humanities and social sciences subjects guide different peer interaction patterns. These differences create distinct competitive and collaborative atmospheres among peers, ultimately impacting students’ Learning Engagement.

## Research Contributions

1. One of the significant contributions of this research is the substantial enhancement and expansion of the Unified Theory of Acceptance and Use of Technology (UTAUT) model’s application in the flipped classroom environment, providing crucial theoretical and practical insights to the field of education. By integrating variables such as performance expectancy, effort expectancy, and peer influence, and incorporating learning engagement and learning capability into the model, this research innovatively constructs an improved UTAUT model. This not only verifies the direct impact of these variables on learning capability but also reveals the mediating role of learning engagement. The research underscores the importance of performance expectancy, effort expectancy, and peer influence in promoting deep learning, active participation, and enhancing learning capabilities, especially in autonomous learning environments like flipped classrooms. These findings offer empirical evidence for understanding and improving flipped classroom design and provide educators with critical information for designing and implementing more effective strategies.
2. The second contribution of this research is demonstrating that learning engagement has a significant positive impact on learning capability, highlighting its central role in students’ learning processes. This finding has important implications for educational practice, providing a basis for improving flipped classroom design and emphasizing the necessity of deep learning engagement.
3. The third contribution lies in exploring how personality and disciplinary backgrounds moderate the effects of peer influence and learning engagement, as well as the impact of performance expectancy on learning engagement. This offers a unique perspective for understanding the influence of individual and disciplinary differences on learning. Furthermore, the model’s innovation lies in its focus on not only the impact of peer influence and performance expectancy on learning engagement but also the moderating effects of personality traits and disciplinary backgrounds. Thus, this research theoretically enhances the empirical foundation of educational psychology and behavioral science, opening new avenues for future educational practice and research. It stimulates research on optimizing learning environments by considering individual and disciplinary characteristics and provides guidance for educators on how to design and improve courses, helping students with different personalities and disciplinary backgrounds better adapt to flipped classrooms.

### Suggestions

#### Strategies for implementing flipped classrooms in practice

Successfully translating flipped classroom research findings into teaching practice is a gradual process. This process requires teachers to comprehensively and deeply understand the teaching philosophy and methods of the flipped classroom, fully recognizing its significant advantages in enhancing students’ learning initiative and deep engagement. Subsequently, based on specific course content and students’ actual needs, teachers should carefully plan and create preview videos aimed at stimulating students’ curiosity and effectively imparting core knowledge. In the classroom environment, teachers should create diversified interactive learning activities, such as group discussions, role-playing, or experimental operations, aimed at promoting students’ internalization and application of the learned knowledge. Simultaneously, establishing an efficient feedback system is crucial to enable teachers to grasp students’ learning progress and encountered problems in real-time, providing precise guidance and support. This series of processes smoothly transitions the research results of the flipped classroom into practical teaching strategies, thereby significantly improving teaching quality and optimizing students’ learning outcomes.

#### Adjustment and optimization of flipped classrooms in different educational environments

The research findings of flipped classrooms have profoundly impacted instructional design, emphasizing student-centeredness and focusing on students’ active learning and collaborative inquiry. In different educational backgrounds, the teaching strategies of flipped classrooms need to be adjusted accordingly to adapt to specific teaching environments and student needs. In the basic education stage, where students’ autonomous learning ability is relatively weak, teachers can design more guiding and interesting preview videos. Meanwhile, teacher-student interaction should be strengthened in the classroom to help students better understand and master knowledge. In contrast, in the higher education stage, where students possess stronger autonomous learning and inquiry abilities, teachers can set more challenging and research-oriented preview tasks and classroom activities to stimulate students’ innovative thinking and critical reflection. Through such adjustment strategies, the teaching model of the flipped classroom can be optimized to maximize its teaching effectiveness in different educational backgrounds.

#### Policy suggestions

The effective implementation of flipped classrooms relies on advanced educational technology platforms, which directly provides evidence for government investment in educational informatization. Based on this, the government can more targetedly increase support for educational technology innovation, thereby promoting the growth and progress of related industries. Additionally, the emphasis on students’ autonomous learning and collaborative inquiry in flipped classrooms aligns with the concept of quality education advocated in current educational reforms. Therefore, the government can formulate corresponding policies based on this, actively encouraging and guiding schools to explore and practice innovative teaching methods such as flipped classrooms. Moreover, educational equity is also a crucial aspect that cannot be ignored. The government should strive to ensure that all students have equal access to high-quality educational resources and technical support. Through this series of comprehensive and logical policy formulation and implementation, the application of educational technologies such as flipped classrooms in teaching will be more widely promoted and developed in-depth.

## Research limitations and future research directions

### Research limitations

1. Sample selection: this research randomly selected 450 students as samples from three universities in Jiangxi Province: Jiangxi University of Finance and Economics, Nanchang University, and Jiangxi Normal University. Although this sample size has a certain degree of representativeness, it is still relatively limited and may not fully reflect the actual situation of all college students. Additionally, the sample only comes from universities in Jiangxi Province, and geographical restrictions may affect the universality of the results.
2. Potential self-selection bias: students willing to participate in the questionnaire survey may have stronger concerns and interests in issues related to learning engagement and learning capabilities. This may cause the sample to deviate from the overall distribution to some extent.
3. Limitations of result interpretation: this research mainly draws conclusions based on questionnaire surveys and structural equation modeling analysis. However, questionnaire surveys inherently rely on respondents’ self-reports, which may be subject to subjective bias or memory reconstruction.

#### Future research directions

1. Expanding sample scope and diversity: future research can consider selecting more diverse universities nationwide as samples to increase the representativeness and universality of the research. Simultaneously, other student groups besides college students, such as middle school students or graduate students, can also be included.
2. Controlling self-selection bias: to more accurately assess the relationship between learning engagement and learning capabilities, future research can adopt more rigorous sampling methods, such as multi-stage sampling, to further reduce self-selection bias.
3. Deeply exploring influencing factors: this research initially explores the impact of personality and disciplinary differences on learning engagement. Future research can further delve into other potential influencing factors, such as family background, learning environment, teacher support, etc., to more comprehensively reveal the complex relationship between learning engagement and learning capabilities.

## Ethical approval and informed consent

All methods were carried out in accordance with relevant guidelines and regulations. All experimental protocols were approved by the Academic Committee of the School of International Economics and Trade, Jiangxi University of Finance and Economics. Informed consent was obtained from all subjects and/or their legal guardian(s).

## Data Availability

The data that support the findings of this research are available from the corresponding author upon reasonable request.
